# Development of a validated HPLC method for the simultaneous determination of flavonoids in *Cuscuta chinensis* Lam. by ultra-violet detection

**DOI:** 10.1186/2008-2231-20-57

**Published:** 2012-10-16

**Authors:** Homa Hajimehdipoor, Babak Mokhtari kondori, Gholam Reza Amin, Noushin Adib, Hossein Rastegar, Maryam Shekarchi

**Affiliations:** 1 Department of Traditional Pharmacy, School of Traditional Medicine and Traditional Medicine and Materia Medica Research Center, Shahid Beheshti University of Medical Sciences, Tehran, Iran; 2 Department of Pharmacognosy, Faculty of Pharmacy, Tehran University of Medical Sciences, Tehran, Iran; 3 Food and Drug Control Laboratories and Food and Drug Laboratory Research Center, MOH & ME, Tehran, Iran

**Keywords:** *Cuscuta chinensis* Lam., Hyperoside, Rutin, Isorhamnetin, Kaempferol

## Abstract

**Background:**

*Cuscuta* species known as dodder, have been used in traditional medicine of eastern and southern Asian countries as liver and kidney tonic. Flavonoids are considered as the main biologically active constituents in *Cuscuta* plants especially in *C. chinensis* Lam.

**Objective:**

In the present study, a fast, simple and reliable method for the simultaneous determination and quantization of *C. chinensis* flavonols including hyperoside, rutin, isorhamnetin and kaempferol has been developed.

**Materials and methods:**

The chromatographic separation was carried out on a reversed phase ACE 5 C_18_ with eluting at a flow rate of 1 ml/min using a gradient with *O*-phosphoric acid 0.25% : acetonitrile for 42 min. UV spectra were collected across the range of 200–900 nm, extracting 360 nm for the chromatograms. The method was validated according to linearity, selectivity, precision, recovery, LOD and LOQ.

**Results:**

The method was selective for determination of rutin, hyperoside, isorhamnetin and kampferol. The calibration graphs of flavonols were linear with r^2^ > 0.999. RSDs% of intra- and inter-day precisions were found 1.3&3.4 for rutin, 1.5&2.8 for hyperoside, 1.3&3.3 for isorhamnetin and 1.7 & 2.9 for kaempferol which were satisfactory. LODs and LOQs were calculated as 1.73 & 8.19 for rutin, 0.09 & 4.19 for hyperoside, 2.09 & 6.3 for isorhamnetin and 0.18 & 0.56 for kaempferol. The recovery averages of above-mentioned flavonols were 90.3%, 97.4%, 98.7% and 90.0%, respectively.

**Conclusion:**

The simplicity of the method makes it highly valuable for quality control of *C. chinensis* according to quantization of flavonols.

## Introduction

**Figure 1:**
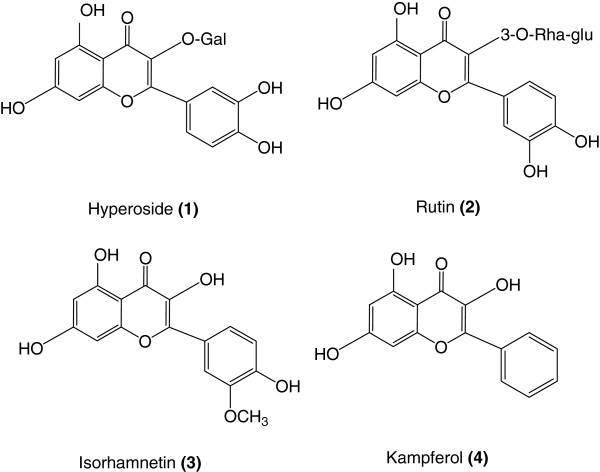
**Chemical structure of flavonols of *C. chinensis.***

*Cuscuta* is a genus consisted of about 100–170 species of yellow, orange, red and rarely green parasitic plants which is the only genus in the family of Cuscutaceae. *Cuscuta* Semen, a crude drug prepared from the seeds of *Cuscuta chinensis* Lam., is commonly used in traditional medicine as a liver and kidney tonic [1]. Many investigations have been established different biological activity of this plant such as improving sexual function [2], anti-cancer [3], immunostimulatory [4-7] and antioxidant activities [7]. The active constituents of the *C. chinensis* are including flavonoids, lignans, quinic acid derivatives and polysaccharides [1,8-10]. These compounds have been suggested to be responsible for the pharmacological activities of the plant [11,12]. Flavonoids, especially rutin, quercetin, isorhamnetin and kampferol are the main biologically active constituents in *C. chinensis* Lam. In addition, these flavonoids have exhibited various pharmacological activities, which to some extent might elucidate the mechanism of clinical effects of this commonly used Chinese medicine. Therefore, their contents can be an important index in quality evaluation of this crude drug. Many fakes were found in the crude drug samples, which seriously influenced the drug’s quality. According to FDA guide line [13], before a plant drug can be legally marketed, its spectroscopic or chromatographic finger prints and chemically assay of characteristic markers are required. Because of the complex nature of a typical botanical drug and the lack of knowledge about its active constituents, the FDA may rely on combination of tests and controls to ensure the identity, purity, quality strength, potency and consistency of these drugs. Hence, quality control of natural drugs is in great demand. Unfortunately, few studies on the quantitative determination of chemical constituents in *C. chinensis* Lam. have been reported so far. In previous papers, total flavonoids and total polysaccharides were determined by colorimetric method, but the results could not reflect the drug’s quality exactly and rapidly [14,15]. In addition, these methods suffered from low resolution and sensitivity. It is known that interaction of multiple chemical compounds contributes to the therapeutics effects of herbal medicines [16]. Therefore, the analysis of multiple components is necessary and helpful to control the quality of herbal medicines. According to our knowledge, there was no report on determination of flavonols and their glycosides in *C. chinensis* Lam. by High Performance Liquid Chromatography (HPLC). So In this paper, the four major flavonols including hyperoside (1), rutin (2), isorhamnetin (3) and kaempferol (4) (Figure 1) in *C. chinensis* Lam. samples were determined simultaneously with a simple, rapid and accurate analysis by reversed phase liquid chromatography.

## Materials and methods

### Plant material

*C.chinensis* was collected from Qazvin-karaj superhighway and identified by Dr. Gh.R. Amin, Herbarium of faculty of Pharmacy, Tehran university of medicinal Sciences, Tehran, Iran, Voucher No.6737 TEH.

### Instrumentation

The HPLC experiment was performed using a Waters Alliance system equipped with a vacuum degasser, quaternary detector. The UV spectra were collected across the range of 200–900 nm, extracting 360 nm for chromatograms. Empower software was utilized for instrument control, data collection and data processing. The column was an ACE C_18_ (4.6 × 250 mm, 5 μm). The mobile phase was a linear gradient with *O*-phosphoric acid 0.25% (A)- acetonitrile (B) for 42 min starting with A:B (95:5) for 2 min, changing to A:B (90:10) for 5 min, A:B (85:15) for 3 min, A:B (80:20) for 13 min, A:B (70:30) for 5 min, A:B (50:50) for 4 min with equilibrating for 10 min. The flow rate was 1 ml/min. The injection volume for all samples and standard solutions was 10 μL.

### Chemicals

Hyperoside, rutin, isorhamnetin and kaempferol standard materials were purchased from ROTH (Karlsruhe, Germany). All solvents were obtained from Merck Co. (Darmstadt, Germany). Water used in all the experiments was deionized by Purelab UHQ Elga.

### Determination of flavonols

#### Solvent effect

The samples were extracted with methanol, acetone and methanol–water 80:20 to determine the effect of the solvent on the extraction efficiency.

#### Optimization of the sample size

To evaluate the effect of the sample size on the accuracy of the flavonoids content estimation, the samples were prepared in triplicate in two sets. In the first set, 0.5 g and in the second set 1 g of powder were weighted and used for extraction.

#### Effect of the extraction method

To determine the effect of the procedure on the extraction, hot solvent extraction (decoction for 30 min), maceration (24 h), and ultrasonic radiation (30 min, three times) methods were compared.

### Sample preparation

Powdered samples (300 μm, 0.5 g) were suspended in 80% methanol (25 ml) and extracted in an ultrasonic bath for 30 min. The suspension was filtered and the remaining powder was extracted two more times using 25 mL 80% methanol. After filtration, the filtrate was transferred to a 100 ml volumetric flask and diluted with the solvent to volume. The obtained solution was filtered through a membrane filter (0.45 μm pore size) prior to injection.

### Preparation of standard solutions

Stock solutions of hyperoside, rutin, isorhamnetin and kaempferol (0.05, 0.2, 0.2 and 0.01 mg/ml) were prepared separately in methanol 80%. Standard multi flavonol solutions were made by using different amount of stock solutions (1–10, 10–60, 10–70 and 0.3-1.6 μg/ml for hyperoside, rutin, isorhamnetin and kaempferol, respectively). Stock and working standard solutions were prepared daily.

### Validation

The reliability of the HPLC-method for analysis of hyperoside, rutin, isorhamnetin and kaempferol was validated through its selectivity, linearity, precision, recovery, limit of detection and limit of quantization [17].

### Selectivity

For the chromatographic method, developing a separation involves demonstrating specificity, which is the ability of the method to accurately measure the analyte response in the presence of all interferences. Therefore, the extraction mixtures obtained from the sample preparation were analyzed and the analyte peaks (flavonols1-4) were evaluated for peak purity and resolution from the nearest eluting peaks.

### Linearity

Due to the verification of the normal distribution of results, linearity was evaluated through the relationship between the concentration of flavonols 1–4 and the absorbances obtained from the UV-HPLC detector. The determination coefficient (*r*^2^) was calculated by means of the least-square analysis [18,19]. The calibration lines were achieved through two replicates of each concentration of hyperoside, rutin, isorhamnetin and kaempferol (1–10, 10–60, 10–70 and 0.3-1.6 μg/mL), to identify the extent of the total variability of the response that could be explained by the linear regression model.

### Precision

The precision of each method indicates the degree of dispersion within a series on the determination of the same sample. Six real samples were analyzed on the same day (intra-day) and three on consecutive days (inter-day), and then the relative standard deviations (RSDs%) were calculated. Each sample was injected to HPLC thrice.

### Recovery

This parameter shows the proximity between the experimental values and the real ones. It ensures that no loss or uptake occurred during the process [18,19]. The determination of this parameter was performed during the method by studying the recovery after a standard addition procedure, with two additional levels. Three replicate amounts of plant (3 × 1.5 g) were weighted and each of them was divided into three equal portions (0.5 g). One part was used as the real sample and others had been spiked with multi-flavonol standard solution containing hyperoside (1&3 μg/mL), rutin (5&10 μg/ mL), isorhamnetin (5&10 μg/mL) and kaempferol (0.2&0.4 μg/mL) in two levels. In each additional level, three determinations were carried out and the recovery percentage was calculated in every case. Each sample was injected into HPLC three times.

## Results and discussions

One of the challenging aspects of method development in quantitative analysis is the complexity of the analysis methods. The best method is the simplest one which could be conducted by different operators and in different labs. However other parameters of a quantitative method such as accuracy and precision demand more complex processes.

### Extraction procedures

Extraction is the main step for the recovery and isolation of bioactive compounds from plant materials, before analysis. It is influenced by chemical nature of compounds, the extraction method employed, sample particle size, as well as the presence of interfering substances. Commonly used extraction solvents for flavonoids are alcohols (methanol, ethanol), acetone, diethyl ether, and ethyl acetate. In our experiment, very polar flavonol glycosides could not be extracted completely with pure organic solvents so 80:20 methanol–water mixture was an excellent choice. Besides the high recovery, less interfering in comparison with other solvents made it a suitable solvent for extraction of all flavonoids and preparation of standard materials. Among different methods of extraction, the ultrasonic radiation was selected in comparison with maceration and hot solvent extraction. Comparison between two experiments showed that the smaller sample size appeared to have a significant effect on the accuracy of flavonols analysis. In general, using ultrasonic radiation of the plant (0.5 g, 30 min, three times) with methanol 80% was selected as the best method for hyperoside, rutin, isorhamnetin and kaempferol analysis (Table 1).

**Table 1:** **The effect of different extraction procedures on recovery of *C. chinensis *flavonols**

**Compound**	**Extraction method**			**Solvent**			**Sample size**	
	***Decoction***	***Maceration***	***Ultrasonication***	***Acetone***	***Methanol***	***Methanol 80%***	***0.5 g***	***1.0 g***
**Rutin**	30.11 ± 1.22^*^	35.23 ± 2.23	42.55 ± 0.54	21.02 ± 1.5	31.31 ± 0.41	42.55 ± 0.54	42.55 ± 0.54	35.3 ± 5.2
**Hyperoside**	3.12 ± 0.21	4.02 ± 0.12	6.01 ± 0.09	4.15 ± 0.22	5.23 ± 0.04	6.01 ± 0.09	6.01 ± 0.09	5.21 ± 1.2
**Isorhamnetin**	23.21 ± 0.18	25.30 ± 0.17	33.49 ± 0.45	34.1 ± 2.3	35.2 ± 0.25	33.49 ± 0.45	33.49 ± 0.45	34.16 ± 6.2
**Kaempferol**	0.26 ± 0.01	0.22 ± 0.02	0.46 ± 0.01	0.49 ± 0.02	0.45 ± 0.03	0.46 ± 0.01	0.46 ± 0.01	0.45 ± 0.03

*: Represents as μg/mL.

### Method development and validation

**Figure 2:**
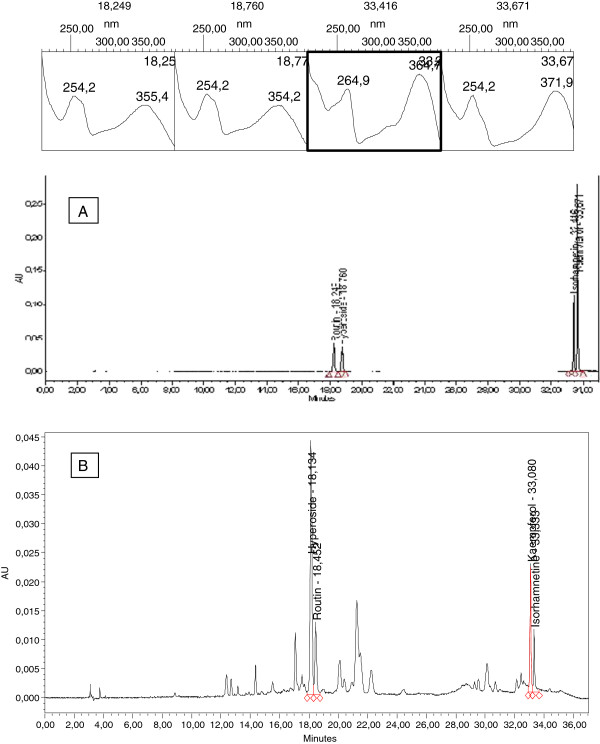
**HPLC chromatogram of A: rutin, hyperoside, kampferol and isorhamnetin standard solution and B: *C. chinensis *sample with chromatographic UV spectra at 200–400 nm.**

As it is shown in Figure 1, all compounds1-4 were tri- or tetra-hydroxylated flavonols, with similar structures, especially in the case of hyperoside and rutin; therefore, it was difficult to separate all components simultaneously. After comparison between the different columns such as C_8_, C_18_, CN and phenyl, the best separation efficiency was obtained by using the C_18_ column. The mobile phase investigations showed that the ratio of organic modifiers, such as the acetonitrile or methanol in the mobile phase, was the key to a good separation. The pH value played an important role in the solute ionization. In order to minimize flavonols ionization, using an acidic mobile phase was obligated. According to this, the best separation was achieved by using 0.25% *O*-phosphoric acid solution. The gradient mode of the instrument was changed to obtain the best resolution and the shortest run time. Each flavonol peak was resolved from the neighboring peaks and displayed excellent peak symmetry and separation efficiency as seen in Figure 2. These groups of compounds had a special chromophoric nature, which made them easy to identify from their UV diode-array absorption spectra. The results obtained from the method validation according to linearity, selectivity, accuracy and precision showed that the proposed method was suitable for the analysis of all four flavonols 1–4. Comparison between the purity threshold and purity angle reported in the empower software showed that the method was specific for hyperoside, rutin, isorhamnetin and kaempferol and the reported peaks were completely separated from the other interfering compounds. The linear relationship between the detector response and different concentrations of flavonols were confirmed as it was shown in Table 2. The relative standard deviations (RSDs%) of the intra-day and inter-day have been shown in Table 3. The results of intermediate precision using different analysts, different instruments, and on different days, showed that these parameters did not have any significant effect on the variation of results (data did not show). After these validation studies, the method’s ability to provide good quantization in our laboratory was confirmed. The last step in the measurement of precision (reproducibility), which focused more on the bias in results, rather than on determining the differences in precision alone, as inter-laboratory crossover studies, would be our next target. Accuracy, which was evaluated as recovery, after spiking the plant samples with standards at two concentration levels have been shown in Table 4.

**Table 2:** **Linearity, LOD and LOQ parameters of flavonols analysis in *C. chinensis***

**Flavonols**	LOD*	LOQ*	**r square**	**Equation**	Linear range*
**Rutin**	1.73	8.19	0.9990	Y = 8752x + 8764	10-60
**Hyperoside**	0.09	4.19	0.9997	Y = 17073x + 2748.7	1-10
**Isorhamnetin**	2.90	6.33	0.9994	Y = 3553.8x + 1305	10-70
**Kaempferol**	0.18	0.56	0.9990	Y = 26807x + 67263	0.3-1.6

*represents as μg/mL.

**Table 3:** **Repeatability of flavonols analysis in *C. chinensis***

**Flavonols**	Mean ± SD^*^(Intra-day n = 6)	**RSD% (Intra-day)**	**RSD% (Inter-day n = 3)**
**Rutin**	42.55 ± 0.54	1.3	3.4
**Hyperoside**	6.01 ± 0.09	1.5	2.8
**Isorhamnetin**	33.49 ± 0.45	1.3	3.3
**Kaempferol**	0.46 ± 0.01	1.7	2.9

*: Represents as μg/mL.

**Table 4:** **Recovery of flavonols analysis in *C. chinensis***

**Flavonols**	^*^Spiked	Found^*^	% Mean recovery^**^	**Total recovery ± SD**
**Rutin**	0.0	42.11 ± 0.71	-	90.3 ± 6.0
	5.0	46.73 ± 0.36	92.4 ± 7.2	
	10.0	50.91 ± 0.60	88.7 ± 5.1	
**Hyperoside**	0.0	5.80 ± 0.12	-	97.4 ± 9.1
	1.0	6.84 ± 0.10	103.0 ± 9.8	
	3.0	8.67 ± 0.20	91.7 ± 6.6	
**Isorhamnetin**	0.0	32.51 ± 0.68	-	98.7 ± 7.9
	5.0	37.62 ± 0.56	100.4 ± 10.9	
	10.0	42.35 ± 0.56	97.0 ± 5.5	
**Kaempferol**	0.0	0.45 ± 0.01	-	90.0 ± 4.1
	0.2	0.63 ± 0.01	90.0 ± 5.1	
	0.4	0.82 ± 0.02	92.5 ± 4.0	

*Represents as μg/mL, ** n = 3.

As it was reported in Table 4, the careful optimization of extraction conditions caused the good recovery for each flavonols 1–4. So this method because of reaching suitable recovery and good precision can be recommended for the quantification of hyperoside, rutin, isorhamnetin and kaempferol in *C. chinensis.*

## Conclusions

This work proposes a new method for simultaneous separating and determining of four flavonols of hyperoside, rutin, isorhamnetin and kaempferol. The most relevant advantage of the proposed method is the simultaneous determination of the four major flavonols in *C. chinensis* Lam. in order to reduce time required for quantitative extraction and analysis. It is a simple, fast, accurate and reliable technique in both chromatographic condition and sample preparation with minimum use of solvents. This method is suitable for quality control of *C. chinensis* Lam. and could be candidate as a routine method in quality control laboratories.

## Competing interests

The authors declare that they have no competing interests.

## Authors’ contributions

HH carried out identification and separation of flavonols from plant. BMK prepared sample and standard solutions. GRA participated in collection and identification of the plant. NA collaborated in validation of the method. HR collaborated in validation of the method. MSh carried out method development and designing of validation protocol. All authors read and approved the final manuscript.
